# The Interplay between Circulating Tumor Cells and the Immune System: From Immune Escape to Cancer Immunotherapy

**DOI:** 10.3390/diagnostics8030059

**Published:** 2018-08-30

**Authors:** Kevin Leone, Cristina Poggiana, Rita Zamarchi

**Affiliations:** Veneto Institute of Oncology IOV—IRCCS, Padua, Italy; kevin.leone@iov.veneto.it (K.L.); cristinapoggiana@gmail.com (C.P.)

**Keywords:** circulating tumor cells, immune system, immunotherapy, cancer biomarkers, liquid biopsy, metastasis

## Abstract

Circulating tumor cells (CTCs) have aroused increasing interest not only in mechanistic studies of metastasis, but also for translational applications, such as patient monitoring, treatment choice, and treatment change due to tumor resistance. In this review, we will assess the state of the art about the study of the interactions between CTCs and the immune system. We intend to analyze the impact that the cells of the immune system have in limiting or promoting the metastatic capability of CTCs. To this purpose, we will examine studies that correlate CTCs, immune cells, and patient prognosis, and we will also discuss relevant animal models that have contributed to the understanding of the mechanisms of immune-mediated metastasis. We will then consider some studies in which CTCs seem to play a promising role in monitoring cancer patients during immunotherapy regimens. We believe that, from an accurate and profound knowledge of the interactions between CTCs and the immune system, new immunotherapeutic strategies against cancer might emerge in the future.

## 1. Introduction

In malignant evolution, cancer cells acquire the capability to invade healthy tissues and colonize distant organs. This process, indicated as metastatic cascade, includes an ordinate series of events involving tumor cells, namely their invasion into surrounding normal tissues, intravasation in the bloodstream, arrest, and extravasation through vascular walls into secondary sites, formation of microscopic colonies, and final proliferation into overt, clinically detectable metastases [1].

Although metastasis accounts for a remarkable 90% of cancer-associated deaths, it remains a poorly understood process because of the complex interplay that the primary tumor establishes with stromal cells, often based on redundant and still unclear signaling pathways [1]. Among stromal cells, those of the immune system especially affect the outcome of tumor progression and metastasis.

Furthermore, it is not yet clear when the metastatic capability of a tumor appears, because direct and indirect evidence have contrasted the view that the spreading of tumor cells to secondary sites represents a late event in tumorigenesis. For example, 30.6% of breast cancer (BC) patients at diagnosis show micrometastases in the bone marrow, independently of disease stage [2], and prostate cancer cells disseminate early [3]. Moreover, karyotypic abnormalities of micrometastases in the bone marrow from BC patients, as well as from animal models, indicate that tumor cell dissemination occurs in the pre-invasive stage of the disease [4]. Consequently, characterization of the primary tumor may not be enough to assess the risk for disease recurrence and to choose the best treatment, while by investigating metastases, which represent the final result of the process, we might lose relevant information about the characteristics necessary to overcome one or more steps of the metastatic cascade.

On these bases, the study of circulating tumor cells (CTCs) has elicited increasing interest, especially after the advent of reliable technologies that permit isolation, quantification, and characterization of tumor cells after intravasation in the peripheral blood and before extravasation at secondary sites. Although this is only an intermediate step of the full metastatic process, it is a crucial step as demonstrated by the prognostic value of CTC numbers [5] and it is thought to contribute to the selection of phenotypic and biological properties that are necessary for tumor cells to complete the whole process [6]. Furthermore, since we can obtain tumor cells from a simple blood draw of a cancer patient as often as necessary, CTCs are considered of utmost importance not only in mechanistic studies of metastasis, but also for translational applications such as patient monitoring, treatment choice, and, in the case of ongoing resistance, treatment change.

For this reason, being aware of the novelty of immunotherapy of tumors in the precision medicine era, we thought to review what we know concerning the relationship between CTCs and the different cell populations of the immune system, with the aim to understand how the latter can prevent or even support the metastatic dissemination of the former.

Our review does not aim to debate different scenarios about the interactions between CTCs and the immune system, with the final goal of supporting one or the other, since the scientific knowledge on this topic is not yet complete. Rather, we feel the need to describe the state of the art regarding what we know on the role and mechanisms of the different immune cell populations in limiting or promoting the dissemination and persistence of CTCs.

To this purpose, in our literature searches, we used the keyword ‘CTC’ (or the full form ‘circulating tumor cell’) in conjunction with ‘macrophages’, ‘dendritic cells’, and so on, according to the paragraph titles. For the keyword ‘immunotherapy’, we combined it with ‘CTC’ and, in some cases, with a third keyword, e.g., ‘biomarkers’, ‘targets’, and ‘PD-L1’.

We reviewed not only studies performed in cancer patients that correlate CTCs, immune system cells, and prognosis, but also studies presenting relevant animal models that, by studying experimental tumors induced by cancer cell lines, or experimental metastasis induced by injection of cancer cell lines in the peripheral blood, which contributed to the understanding of immune-mediated mechanisms of metastasis. Based on our selection criteria, we used the acronym ‘CTC’ only when talking about studies on human samples or on animal models in which CTCs were shed from primary tumors. In all other cases (in vitro assays and in vivo experimental metastasis), we talked about ‘tumor cells’ since in these conditions neoplastic cells were not actually circulating or part of a real metastatic process.

We then reviewed papers reporting results on immunotherapies, with particular regard to what we can directly monitor in cancer patients by studying CTCs.

Finally, we briefly looked at future opportunities, i.e., new immunotherapeutic strategies that we could implement using the knowledge on metastatic mechanisms gained from studies on CTCs.

## 2. Immune-Surveillance and Immune-Support of CTCs 

The success rate of metastasis is low since only a few of the thousands of CTCs daily released in the bloodstream survive and form secondary lesions. For instance, in murine experimental metastasis models, Fidler found that only 1% of tumor cells survived in premetastatic lungs 24 h after intravenous injection [7], and Luzzi et al., at day 13 post-injection, observed that only 1% of liver micrometastases progressed to form macrometastases and that 36% of tumor cells remained isolated; 95% of which were dormant (2% proliferative and 3% apoptotic) [8]. Several reasons can explain this inefficiency, including the mechanical stress in blood vessels, the complexity of the translocation from one site to another, the presence of a final microenvironment unsuitable for proliferation, and the intervention of host immune surveillance [7,8,9].

In principle, we cannot exclude that the relationships between the immune system and tumor cells differ between primary tumor and peripheral blood. Indeed, in peripheral blood, CTCs migrate as single cells or tumor microemboli and undergo changes that are expected to modify their phenotype [10], so that CTCs might continue or interrupt their crosstalk with the immune system. Furthermore, after intravasation, CTCs lose all the interactions with both the extracellular matrix (ECM) and stromal cells; in this situation, cytokines and other soluble factors produced by immune system cells, which are usually only locally active, might no longer be effective on CTCs. In any case, it is conceivable (and generally accepted) that CTCs, leaving the protected microenvironment of the primary tumor, encounter further immune surveillance in nontumor tissues.

Immune cells ambiguously show both anti- and protumor effects. They can promote tumor progression and metastasis by creating an immunosuppressive, tolerogenic context or mediating ECM degradation and angiogenesis [11,12,13]. Tumor-derived soluble factors (TDSFs) mediate the recruitment of myeloid cells and hematopoietic progenitors in the target organ to adapt local microenvironment for CTC homing [14]. For example, tumor necrosis factor (TNF)-α supports survival and proliferation of tumor cells and increase vascular permeability [15,16]. Also, immunohistochemistry on biopsies from BC patients revealed that the chemokine receptor CXCR4 favors tumor cell homing in the bone marrow [17].

Interestingly, when Hensler and colleagues compared BC patients and healthy donors for gene expression profiles in both CTCs (obtained by immuno-magnetic sorting) and peripheral blood mononuclear cells (PBMCs), they found a higher expression of metastasis-related genes, such as those for ECM degradation and epithelial–mesenchymal transition (EMT), in patient PBMCs [18].

**Figure 1:**
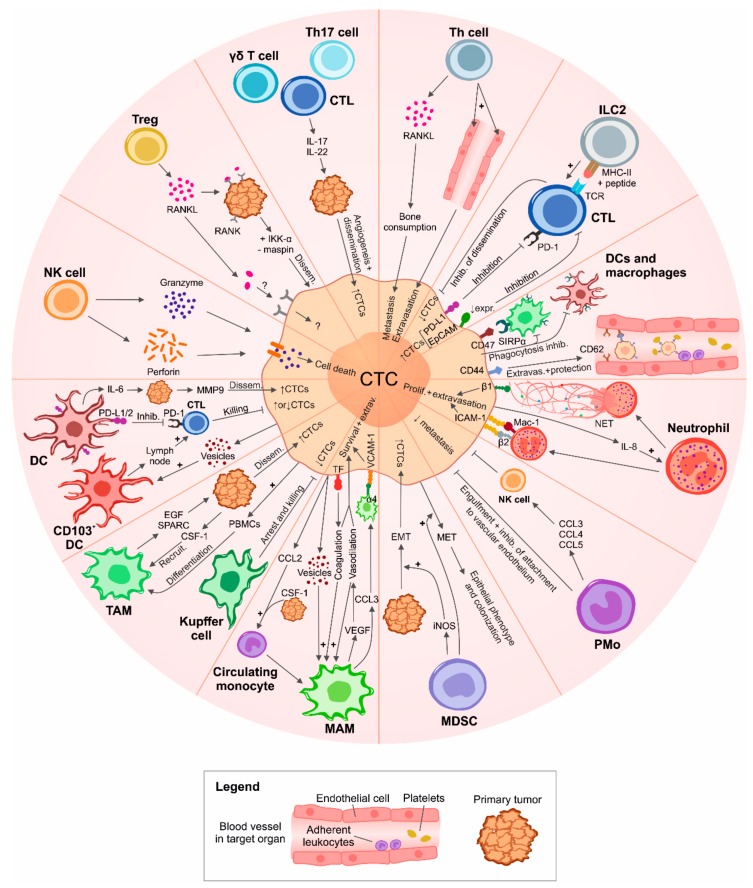
Representation of the main known mechanisms involving an interplay between CTCs and anti- or protumor cell populations of the immune system (+: induction).

In the subsections below, we will consider the most important infiltrating and circulating immune cells that hinder or favor the dissemination of CTCs, in some cases directly interacting with them. The main mechanisms are depicted in Figure 1. 

### 2.1. Natural Killer (NK) Cells

The number of circulating NK cells has been observed to increase in metastatic breast, colorectal, and prostate cancer patients as compared to healthy donors [19]. NK cells can intercept CTCs in the bloodstream and destroy them before extravasation, thus preventing metastasis [20]. A study performed on mice injected with human colon cancer cells tried to elucidate the mechanisms used by NK cells to kill cancer cells. Direct perforin-dependent killing resulted more effective than indirect killing with apoptosis-inducing factors, since the former significantly delayed primary tumor growth, reduced the number of CTCs by 80% as assessed by real-time PCR, and hindered the formation of metastases, with respect to the latter [21].

Accordingly, low NK cell activity is associated with advanced disease and metastasis [22,23]. Metastatic BC patients with >5 CTCs per 7.5 mL of blood had circulating NK cells showing deficient lytic ability in chromium-51 release assays as compared to those from patients with ≤5 CTCs, and an inverse correlation between CTCs and progression-free survival (PFS) was found [24]. Also in metastatic breast, colorectal, and prostate cancer patients, the cytotoxic activity of NK cells inversely correlated with the level of CTCs detected by the CellSearch system (CS, Menarini-Silicon Biosystems, Castel Maggiore (BO), Italy) [19].

### 2.2. CD8^+^ and CD4^+^ T Cells

Our current knowledge concerning the function of CD8^+^ cytotoxic T lymphocytes (CTLs) and CD4^+^ T helper (Th) cells in immune surveillance of CTCs is very limited. Their infiltration in primary tumors, together with that of NK cells, correlates with an increase of disease-free survival (DFS) and overall survival (OS), and a reduction of metastasis and relapse [25,26]. Accordingly, in patients with metastatic BC, low circulating lymphocyte levels and high CTC levels, as assessed by CS, were found to be independent poor predictive factors for PFS and OS [27]. In inflammatory BC patients, the presence of CS-detected CTCs correlated with a reduction of CD3^+^ T cells, CD4^+^ T cells, and CD8^+^ T cells synthesizing TNF-α and IFN-γ [28]. In stage IV non-small-cell lung cancer (NSCLC) patients, Ye et al. showed that the number of CTCs as determined by SET-iFISH correlated negatively with that of NK cells and CD3^+^, CD4^+^, and CD4^+^/CD8^+^ lymphocytes, while positively with metastasis [29]. Another study performed on late stage NSCLC patients gave comparable results, since CTCs showing both epithelial and mesenchymal markers in RNA-ISH assays negatively correlated with CD3^+^ and CD8^+^ T cells; moreover, CTC levels positively correlated with metastasis and a worse clinical outcome [30].

In a different study, BC patients positive for CTCs after AdnaTest were characterized by a significant increase of peripheral first apoptosis signal receptor (FAS)-positive Th cells [31]. The induction of apoptosis in T cells could represent an escape mechanism used by CTCs (see Section 3) and potentially explain the observed CTC-related lymphocytopenia. However, it is not clear if CTCs control the number of lymphocytes or if the increase in CTCs is the consequence of a lymphocyte dysfunction due to other factors, in particular, immunosuppression exerted by the primary tumor. 

### 2.3. Regulatory T Lymphocytes (Tregs)

By releasing TDSFs, such as interleukin (IL)-10, transforming growth factor (TGF)-β, and galectin 1, the tumor can stimulate the generation and expansion of Tregs, which can impair antitumoral immune responses, subsequently allowing disease progression and metastasis [32,33]. Indeed, in metastatic melanoma patients, an increased frequency of Tregs correlated with disease progression [34], while in node-negative BC patients, it correlated with both disease progression and lymph node micrometastases [35]. Several studies on different murine tumor models closely associated Tregs with the establishment of metastases [33,36,37,38,39].

Accordingly, in metastatic carcinoma patients, circulating Tregs were more abundant as compared to healthy donors, although the numbers of Tregs did not correlate with CTCs; probably due to the low-sensitivity test (immunocytochemistry) used to identify CTCs, as concluded by the authors themselves [40]. Conversely, in inflammatory BC patients, CS-enumerated CTCs positively correlated with circulating Tregs [28]. In the already-mentioned work by Ye et al. performed on advanced NSCLC patients, high numbers of CTCs correlated, not only with lymphocytopenia and metastasis, but also with high numbers of Tregs [29]. In addition, in a study performed with PCR and FACS, high pre-resection levels of both epithelial cell adhesion molecule (EpCAM)-positive CTCs and circulating Tregs correlated with a higher risk of post-resection recurrence and metastasis in hepatocellular carcinoma patients [41]. Thus, the combination of CTCs and circulating Tregs might provide a novel prognostic predictor of cancer progression. 

Interestingly, in a transplantable BC mouse model, Tregs infiltrating the primary tumor were found to produce receptor activator of nuclear factor kappa-B ligand (RANKL), a tumor-necrosis-factor family member involved in bone regeneration and remodeling, as well as in mammary gland hyperplasia during pregnancy. Released RANKL stimulated lung metastatic dissemination of RANK^+^ tumor cells through activation of IKK-α and downregulation of the metastasis inhibitor maspin [42]. Noteworthy, RANK has been detected on CTCs from melanoma patients [43].

### 2.4. Other Lymphocytic Subsets

In an invasive BC model, the tumor-released chemokine CCL2 and the subsequent IL-1β release from TAMs (see Section 2.8) were shown to induce IL-17 production in γδ T cells. In turn, IL-17 caused a systemic granulocyte colony-stimulating factor (G-CSF)-dependent expansion of neutrophils, that suppressed CTLs and led to increased metastasis [44,45]. CD4^+^ T helper 17 (Th17) cells also produce IL-17 and have been associated with MDSC-mediated immunosuppression (see Section 2.7) and metastasis [46]. Accordingly, in a study on a transplantable murine colorectal cancer (CRC) model, the authors found corresponding changes between serum IL-17A levels and the FACS-determined prevalence of CTCs at different stages of disease; moreover, IL-17A promoted angiogenesis and metastasis in vivo, as well as matrix metalloproteinase 9 (MMP9)-dependent invasiveness of tumor cells in vitro [47]. Mego et al. also showed a correlation between CS-derived CTC counts and the percentage of activated IL-17–producing CD8^+^ T cells [28]. Other papers proved that systemic IL-17, through activation of the IL-6/Stat3 pathway and release of MMP2/9, TNF-α, and vascular endothelial factor (VEGF), induces tumor cell migration and metastatic growth in lung cancer-bearing mice [48,49,50]. IL-22 is another cytokine produced by γδ and Th17 cells that induces Stat3 and MMP9 in cancer cells to increase their motility and, in human pancreatic ductal adenocarcinoma, their metastatic capability [51].

Mammary tumor antigen-activated CD4^+^ T cells can prepare the bone premetastatic niche by releasing RANKL and inducing osteolytic bone disease, with subsequent consumption and metastatic colonization of the bone cavity [52]. In another study, allergen-induced pulmonary inflammation was linked to a higher risk of lung experimental metastasis due to a CD4^+^ T cell-mediated activation of the vascular endothelium, which, in microfluidic in vitro assays, was required for an enhanced transendothelial migration of cancer cells [53].

In a recent study on spontaneous and transplantable tumor-bearing mice, the tumor-evoked regulatory B cells (tBregs), a lymphocyte subpopulation that derives from TDSF-conditioned B cells, induced the differentiation of CD4^+^ T cells into Tregs and fully activated the prometastatic functions of cancer-primed MDSCs through the TGF-β signaling, eventually increasing metastasis [36,54]. 

Finally, type 2 innate lymphoid cells (ILC2s) are gaining increasing interest as they can activate myeloid cells by secreting cytokines and directly induce T cells by expressing MHCII molecules [55]. ILC2s participate in immune surveillance against CTCs, since in transplantable murine models they are recruited to the primary tumor in an IL-33-reliant manner and mediate CTL activation together with DCs, hence producing a decrease in tumor growth, CTCs (evaluated by FACS), and metastasis [56]. 

### 2.5. Neutrophils

The primary tumor can secrete G-CSF, which induces granulopoiesis in the bone marrow and recruits neutrophils—cells actively involved in tumor progression and metastasis [57,58]. In spontaneous and transplantable BC mouse models, neutrophils were shown to accumulate in premetastatic lungs and produce H_2_O_2_, with subsequent killing of incoming CTCs (as demonstrated by histology) and inhibition of metastasis; the authors also stated (without presenting relative data) that the number of CTCs was not affected by neutrophils [59]. Nonetheless, the surveillance exerted by neutrophils over CTCs is so far largely unexplored.

Conversely, more data are available on neutrophil support to CTCs. As already discussed, systemically raised G-CSF upon γδ T cell activation polarizes neutrophils towards an immunosuppressive behavior that favors metastatic progression [44,45]. Moreover, G-CSF also induces homing of Ly6G^+^ Ly6C^+^ granulocytes in the premetastatic lungs of tumor-bearing mice, where they can release the Bv8 protein that induces angiogenesis, further mobilization of myeloid and tumor cells, and final metastasis [58].

Neutrophils have been extensively associated to tumor angiogenesis and, thus, to an easier dissemination of tumor cells [60]. For example, UV irradiation-induced metastasis of primary melanoma was shown to be mediated by activated neutrophils that prompt angiogenesis and TNF-dependent migration of melanoma cells towards vascular endothelial cells, both in vitro and in vivo [61].

Several in vivo imaging studies showed that CTCs colocalize with endothelium-bound neutrophils in the premetastatic vascular network, suggesting that neutrophils can retain cancer cells and facilitate their extravasation. For example, adherent neutrophils within the inflamed liver sinusoids interact with intravenously-injected lung cancer cells and increase their tethering ability to CD62 (selectin) on the vascular endothelium [62,63]. In vitro assays and in vivo experimental metastasis demonstrated that melanoma cells entrapped in premetastatic lung capillaries release IL-8 and recruit neutrophils that, in turn, upregulate β2 integrins and bind intercellular adhesion molecule (ICAM)-1 on tumor cells, favoring their anchoring to blood vessels [64]. Activated neutrophils also adhere to CTCs through interaction of Mac-1 on the former and ICAM-1 on the latter [63]. ICAM-1 is then able to trigger migration-related signaling pathways inside tumor cells, thus promoting extravasation and colonization of adjacent tissues [65].

Furthermore, once stimulated by G-CSF, IL-8, or other pro-inflammatory factors, neutrophils undergo a process called NETosis and release neutrophil extracellular traps (NETs), i.e., networks of DNA and antimicrobial proteins, which, in physiological conditions, catch and contain pathogens. During systemic inflammation in mice, NETs capture systemically-delivered cancer cells and improve their adherence to liver blood vessels, extravasation, and metastatic spreading [66]. NET-released high mobility group box 1 (HMGB1) augments cancer cell adhesion, proliferation, and migratory capabilities in vitro in a toll-like receptor 9 (TLR9)-dependent manner [67]. Remarkably, in a transplantable tumor model, CTCs themselves induced the formation of metastasis-supporting NETs in the absence of infection [68], and this could depend on neutrophil priming upon tumor release of G-CSF [69]. In a recent study performed on a murine intra-abdominal sepsis model that mimics postoperative inflammation, Najmeh and colleagues showed the central role of β1 integrin in mediating the interaction between CTCs and NETs [70].

In metastatic CRC patients who had undergone liver resection, postsurgical inflammation caused an increase in NETs, which correlated with a >4-fold metastasis-dependent reduction in DFS [67]. NETs have also been observed in patients with pancreatic ductal adenocarcinoma [71] and BC [68], while in gastric cancer patients the number of NETs resulted higher than in healthy donors and increased with disease progression [72]. However, to date, no findings are available for NETs and CTCs in human cancer.

### 2.6. Monocytes

Circulating monocytes comprise two subpopulations, classical and nonclassical monocytes. As we will discuss later, classical ‘inflammatory’ monocytes can extravasate and differentiate into macrophages with protumor and prometastatic functions. Conversely, in response to the CX3CL1 chemokine, nonclassical ‘patrolling’ monocytes (PMos) accumulate in capillaries, where they clear circulating cellular debris, and intervene in inflammation with a protective role [73]. Hanna et al. showed in different murine metastatic tumor models that, up to 24 h after intravenous cancer cell injection, PMos are recruited to premetastatic lung capillaries through the CX3CL1/CX3CR1 axis and engulf tumor material, while as early as 4 h after injection they interact with tumor cells in circulation and hamper their attachment to the lung microvasculature. Moreover, the PMo-secreted CCL3, CCL4, and CCL5 chemokines summon and activate NK cells, thus leading to further elimination of metastasizing tumor cells and prevention of lung metastasis [74].

Finally, the expression of TLR2 and TLR4 on whole, peripheral monocytes was found to inversely correlate with the level of CS-detected CTCs in metastatic breast, colorectal, and prostate cancer patients [19]. 

### 2.7. Myeloid-Derived Suppressor Cells (MDSCs)

MDSCs are heterogeneous, immature myeloid cells comprising a polymorphonuclear subset (PMN-MDSCs) and a monocytic subset (M-MDSCs), which can be respectively distinguished from granulocytes and monocytes due to their high immunosuppressive activity [75]. Infiltrating and circulating MDSCs can favor metastasis by creating a tolerogenic microenvironment in both the primary tumor and metastatic sites [54,76,77,78]. Interestingly, in a study on portal vein blood samples from pancreatic cancer patients, the authors found a correlation between numbers of circulating M-MDSCs and active FACS-isolated K-RASmut^mRNA+^ CTCs, suggesting that the establishment of liver metastases in these subjects may be supported by immunosuppression-dependent CTC survival in the bloodstream [79].

Furthermore, MDSCs can directly stimulate aggressiveness of tumor cells. By releasing IL-6, MDSCs elicit STAT3 activation and invasive capabilities of BC cells, with subsequent increase in tumor and metastasis burden [80]. MDSCs can also facilitate neoplastic cell dissemination by releasing MMP9 and degrading the ECM [81], as well as by upregulating MMP2, MMP13, and MMP14 in BC cells [82]. MMP9 from PMN-MDSCs has also been implicated in the generation of an aberrant and leaky vasculature in the premetastatic lung [83]. Additionally, PMN-MDSCs can promote EMT in melanoma cells through activation of the TGF-β, epidermal growth factor (EGF) or hepatocyte growth factor (HGF) signaling pathways, leading to enhanced metastasis [84]. Ouzounova et al. recently showed in a transplantable murine BC model that tumor-infiltrated M-MDSCs promote inducible nitric oxide synthase (iNOS)-mediated EMT and cancer stem cell properties in tumor cells at the invasion frontline; then, PMN-MDSCs in metastatic lungs induce mesenchymal–epithelial transition (MET) in CTCs and restore their original phenotype to foster settlement and proliferation [85]. Thus, MDSCs seem to be involved in EMT, although the role of their two subsets needs to be clarified.

### 2.8. Macrophages

Kupffer cells are liver resident macrophages able to detect and arrest CTCs while passing in the bloodstream and remove metastasizing tumor cells from the hepatic parenchyma [86]. Indeed, CTC counts obtained by CS and Epispot in paired peripheral and mesenteric blood samples from CRC patients suggested that the liver entraps a fraction of CTCs [87]. Kupffer cells may act both by activating adjacent T cells against CTCs and by recognizing opsonized tumor cells and directly killing them [86,88].

Beyond tissue-resident macrophages, those derived from circulating monocytes and M-MDSCs can actively participate in cancer, since tumor-associated macrophages (TAMs) infiltrate advanced tumors [89,90] and their detection correlates with a poor prognosis [91,92,93]. TAMs can directly prompt the migration of tumor cells by secreting paracrine factors. As shown by Wyckoff et al., one of these factors is EGF, which also activates tumor cells to release colony-stimulating factor (CSF-1), thus promoting the motility of TAMs themselves [94]. Loop mechanisms like this explain why TAMs and tumor cells often move together in tumor stroma toward blood vessels [94,95]. Accordingly, the inhibition of CSF-1 or EGF signaling in tumor-bearing mice undergoing intravital imaging experiments reduced metastasis by decreasing the number of macrophages and tumor cells able to leave the primary tumor [94]. Another example is represented by TAM-released secreted protein acidic and rich in cysteine (SPARC), which results to be necessary for metastasis, since it favors migration of cancer cells by aiding their integrin-mediated interaction with nearby stroma [96]. 

TAMs have also been shown to promote tumor cell invasion through release of other factors, namely:

1. Oncogenic miR-22–containing exosomes, able to trigger the Mef2c-β-catenin pathway [97];
2. the chemokine CCL18, which induces calcium signaling and integrin clustering [98];
3. the chemokine CCL20 recognized by the CCR6 receptor [99].

Furthermore, TAMs can facilitate cancer cell migration by activating EMT, for example upon release of lipocalin-2 (LCN2) [100], TNF-α [101], IL-8 [102,103], IL-6 [104], and TGF-β1 [105,106], which operate via activation of different EMT-promoting molecular pathways (e.g., Gas6/Axl-NF-κB, JAK2/STAT3/Snail, and PI3K/Akt). When experimental blocking of these pathways was performed, a reduction in the number of metastases was observed, suggesting the importance of TAMs in tumor cell dissemination and disease progression.

Motility of cancer cells can also be enhanced upon physical interaction with TAMs, which induce both RhoA activity and Notch1 signaling in tumor cells. In turn, Notch1 regulates Mena^INV^ expression, which is required for the formation of invadopodia, matrix degrading protrusions used by cancer cells for invasion, and transendothelial migration [107,108]. Consistently, when mice with BC xenografts were treated with a Notch1-blocking antibody, CTCs (counted as plated, colony-forming cells from blood) diminished when compared to the control group [108].

Another study investigated the interplay between macrophages and CTCs. Hamilton and coworkers first established two permanent CTC lines from blood samples of advanced stage small cell lung cancer patients and, then, cultured in CTC-conditioned media healthy donor-derived PBMCs, which afterwards differentiated into monocytes/macrophages expressing the TAM markers CD14, CD163, and CD68. In addition, macrophage supernatants contained several soluble factors linked to tumor cell invasiveness, angiogenesis, and immune protection, thus suggesting that CTCs might educate TAMs to support their dissemination in vivo [109].

TAMs are not the only macrophages with a metastasis-promoting activity. Circulating monocytes and myeloid progenitors can be chemoattracted by CTC-derived CCL2 into blood vessels of the metastatic organ and, then, can anchor to the endothelium and transmigrate [110]. Once extravasated, these cells can bind CSF-1 released by cancer cells and differentiate into metastasis-associated macrophages (MAMs) [11,111,112]. 

MAMs and other infiltrating myeloid cells can promote vasodilatation in the premetastatic niche by directly secreting VEGF or mediating protease-dependent release of ECM-bound VEGF. Thus, the more abundant blood flow allows further accumulation of MAMs and CTCs [110,112,113]. Another synergistic mechanism of metastatic seeding is based on the tissue factor (TF) expressed on systemically-injected cancer cells, which can recruit platelets and activate coagulation in blood vessels near the target organ; this allows the arrest of other cancer cells and monocytes/macrophages in circulation [114]. Many other recruitment mechanisms are still not fully elucidated, such as that recently reported after intravital imaging in mice by Headley et al. Here, the authors showed that metastasis-promoting CTC-shed 5 µm microparticles and were able to enter neutrophils, monocytes, and macrophages in metastatic lungs within 24 h after arrival of CTCs [115].

In the context of the premetastatic niche, MAMs acquire metastasis-promoting functions. Once activated by CCL2, MAMs produce the autocrine chemokine CCL3, which enhances the interaction between vascular cell adhesion molecule-1 (VCAM-1) on tumor cells and the α4 integrin on MAMs, resulting in a reciprocal, efficient retention and extravasation of macrophages and CTCs at the metastatic site, as demonstrated in various spontaneous and transplantable murine cancer models [116]. In addition, through both VEGF-mediated vasodilatation and physical interaction, MAMs support extravasation, survival, and proliferation of CTCs. Indeed, the elimination of MAMs in metastasis-bearing mice reduces the number of extravasating cells (as determined by real-time PCR) and further metastatic growth [112]. A possible explanation of this survival advantage for cancer cells was proposed by Chen et al., who showed that the binding between α4 integrins on MAMs and VCAM-1 on tumor cells can trigger the Ezrin-PI3K/Akt anti-apoptotic pathway in the latter [117].

Like TAMs in the primary tumor, MAMs in metastatic sites also produce proteases to aid tumor cell invasion, such as cathepsin S, which has been shown to degrade the JAM-B junctional adhesion molecule in the blood–brain barrier and enhance breast–brain metastasis in both murine models and patients [118].

### 2.9. Dendritic Cells (DCs)

DCs comprise the conventional (myeloid) and plasmacytoid (lymphoid) subsets [119]. In both cases, mature DCs are considered to be immunostimulatory and, accordingly, mature DCs that infiltrate the tumor have been associated with a better patient outcome in terms of tumor progression and metastasis [120,121,122]. In BC patients positive for CTCs according to CS, circulating DCs showed increased expression of TLR2, TLR4, and TLR8 and a decreased expression of TLR3 as compared to negative patients. However, it is not clear if this represents an effort of the immune system to respond to tumor-derived ligands or if it is associated to an immune dysfunction potentially having protumor effects [123]. Nonetheless, intravital imaging on metastatic lungs in mice injected with cancer cells showed that resident CD103^+^ DCs—a subset able to cross-present antigens directly to CTLs—can internalize CTC-derived microparticles and migrate to lymph nodes, where they enhance antitumor CTL responses and restrain the metastatic burden [115].

Tumor-infiltrating DCs often do not efficiently stimulate immune responses, since TDSFs can cause accumulation of immature DCs and decreased production of mature DCs inside the tumor, impairing their APC functions [120,124]. Mego et al. recently related the phenotype of circulating conventional and plasmacytoid DCs to both CS CTC counts and clinical outcome in inflammatory BC patients. The authors demonstrated that patients with ≥5 CTCs, as compared to patients with <5 CTCs, had a significantly more advanced disease stage, a worse OS, a reduced percentage of conventional DCs producing TNF-α, IFN-α, and IL-12 and a higher expression of CCR7 and CD86 on conventional and plasmacytoid DCs, respectively. These observations suggest that patients with ≥5 CTCs had defects in DC number and function, despite an enhanced activation and maturation, and a potentially compromised Th1-like immune response [125].

Both DC subsets have also been directly linked to metastasis. CCL2 and LCN2 from tumor cells induce EMT and generation of regulatory dendritic cells (DCregs), DCs that show an immunosuppressive behavior, low expression of the stimulatory molecules HLA-DR, and CD86, and high expression of the immunosuppressive molecule programmed death-ligand 1 (PD-L1). In turn, DCregs activate Tregs and inhibit tumor-specific CTLs, finally enhancing tumor growth and metastasis [126]. 

Another immunosuppressive DC subpopulation has been newly described in a murine pancreatic ductal adenocarcinoma model, where, in response to tumor GM-CSF, monocyte-derived CD11b^+^ CD11c^+^ MHCII^+^ CD24^+^ CD64^low^ F4/80^low^ DCs infiltrated the premetastatic liver. These cells activated Tregs, inhibited CTLs and reduced metastasis through a mechanism involving the MGL2 lectin and PD-L2 [127]. Interestingly, besides impairing DC maturation as mentioned above, tumor-derived exosomes are also able to activate DC prometastatic activity. Indeed, exosomes expressing HSP72 and HSP105 can trigger a TLR2- and TLR4-dependent IL-6 release from conventional DCs and a consequent MMP9 expression in tumor cells with enhanced invasion and metastasis [128]. Furthermore, the number of CD83^+^ mature DCs in the primary tumor of CRC patients correlated with the presence of tumor cells in blood and lymph vessels as assessed by histology, and local lymph node metastases [129].

Likewise, the involvement of plasmacytoid DCs in metastasis has been investigated, although to date few references are available. This subset expands in bone lesions derived from BC and leads to a Th2-like response, as well as to an accumulation of Tregs and MDSCs. Subsequent immunosuppression and release of osteolytic cytokines elicit bone destruction, thus enhancing tumor growth and metastasis [130]. An accumulation of plasmacytoid DCs has also been observed in metastasis from other malignancies, such as BC [131] and melanoma [132]. Moreover, in the peripheral blood from gastric cancer patients, the number of these cells increased in the case of advanced disease and presence of lymph node metastasis [133].

### 2.10. Other Circulating Immune Cells Interacting with CTCs

CTCs can be found as single cells or clusters, named ‘circulating tumor microemboli’ (CTMs), that also comprise leukocytes, endothelial cells, fibroblasts, and other cells held together by cell adhesion proteins [134,135,136]. CTCs inside CTMs are protected from both immune recognition and therapeutics; this ‘stealthiness’ represents a tumor escape mechanism, which can also be provided by platelets alone (see Section 3). In this regard, Jiang at al. recently proposed a microfluidic method to isolate platelet-coated CTCs and CTMs that was not based on the detection of CTC surface epitopes, in this way overcoming the problems associated with masking by platelets and other cells. An intriguing hypothesis suggested by the authors is that platelets may enable CTC-immune cell interactions inside CTMs [137].

A particular a CD14^+^ CD11c^+^ CD45^+^ myeloid subpopulation has been observed inside CTMs. Adams and colleagues named these cells ‘cancer-associated macrophage-like cells’ (CAMLs) and described them as giant cells (30–300 µm in length) with large multiple or polylobated nuclei (14–64 µm in diameter) [138]. The expression of CD14 and CD45 ranges from intense to absent, and the morphology of these cells is very variable, since it can be amorphous, round, oblong, tadpole-, or spindle-shaped. This phenotypic variability, similar to the plasticity of macrophages, suggests that CAMLs may also have different stages of maturation and differentiation [138]. CAMLs have been detected in the peripheral blood of patients with breast, prostate, pancreas and lung cancer in percentages ranging from 81 to 97% of total patients, whilst totally absent in healthy individuals [138,139].

Interestingly, CAMLs can be EpCAM-positive and/or CK8/18/19-positive, although it is still unclear if they directly express these epithelial markers (at different levels depending on the differentiation stage) or if they engulf material of epithelial origin. A possible explanation is that CAMLs internalize tumor cells/CTCs or their debris, as suggested by the presence of tumor-specific markers and mutations inside CAMLs [138]. Accordingly, other researchers working on macrophage–tumor cell fusions (MTFs) could cultivate resulting fused cells present in the blood from melanoma patients and observe primary tumor-specific mutations [140]. The internalization/fusion hypothesis is supported by the fact that CAMLs seem to originate in the primary tumor and increase in blood samples from patients responding to radiotherapy, chemotherapy, or other treatments, generally when dead tumor cells and debris accumulate [138,141]. CAMLs have also been shown to actively interact with CTCs or express CD146 and TIE2, markers that can suggest a pro-angiogenic activity [138]. In support of a protumor role of these cells, in metastatic BC patients, EpCAM^+^ CAMLs correlate with shorter OS and PFS [142].

Therefore, both the origin and function of CAMLs in the tumor context are still under investigation, but they appear to be interesting liquid biopsy-based predictors of tumor activity and response to therapy. It cannot be excluded that CAMLs might also have an active role in helping CTC intravasation, extravasation, or survival in the bloodstream, thus participating in the metastatic process.

## 3. CTC Evasion from Immune Surveillance

Since the early phases of tumor progression, a delicate equilibrium arises between the antitumoral immunity and tumor escape mechanisms. The selection of less immunogenic tumor cell clones (cancer immunoediting) and the promotion of an immunosuppressive microenvironment able to limit immune responses and favor neoplastic progression are paramount in this process [12].

Several mechanisms have been hypothesized through which CTCs could escape or survive from encounters with immune cells [143]. Some mechanisms involve modifications in MHCI molecules:

1. The downregulation or loss of surface MHCI expression to escape the action of CTLs (an event that, in fact, makes them susceptible to the action of NK cells) [144];
2. the acquisition of a ‘pseudonormal’ phenotype by the transfer of MHCI molecules from the surface of platelets to escape NK cell-mediated cytotoxicity [145];
3. the expression of cytokeratins (CK8, CK18, CK19) that interfere with the recognition of MHCI complexes by T cell receptors (TCRs) on CTLs [146].

Other mechanisms not involving MHCI modifications are:

1. The expression of PD-L1, which prevents T cell-mediated destruction [147,148,149,150];
2. the expression of CD47, which provides a ‘don not eat me’ signal [151,152,153];
3. an altered expression of the apoptotic FAS and/or FASL proteins that may induce the apoptosis of T cells [31] or protect tumor cells from FAS-mediated apoptosis [154];
4. the association of CTCs inside CTMs, where they are hidden and protected from immune attacks [155];
5. the interaction with platelets, which induce EMT-like features in CTCs [156], promote their arrest and extravasation [157], and, as already mentioned, form a coating shield that provides them with ‘stealth’ properties and helps their survival in the circulation [158].

### 3.1. CTC Surface Markers Involved in Immune Escape and Metastatic Dissemination

Several CTC surface markers, potentially involved in CTC escape from the immune system, could represent therapeutic targets to prevent metastasis. 

#### 3.1.1. CD44

CD44 is a ubiquitous multistructural and multifunctional cell surface glycoprotein. It participates in a wide variety of cellular functions including cellular adhesion, hyaluronate degradation, lymphocyte activation, lymph node homing, myelopoiesis, lymphopoiesis, angiogenesis, and cytokine release. CD44 is overexpressed in several tumors [159]. The binding of CD44 on migrating tumor cells to CD62 on endothelial cells is responsible for the initial steps of extravasation. Moreover, CD62 is also expressed on platelets and its binding to CD44 creates a coat that protects tumor cells from cytotoxic effector cells in in vivo models [160]. Alternative splicing determines structural and functional diversity of this protein and may be related to tumor metastasis [161].

CTCs from patients with metastatic BC, capable of metastasizing in immunocompromised mice, express CD44 [152]. Katoh et al. collected CTCs from 150 patients affected by sporadic CRC and examined the relationship between expression of the CD44v9 mRNA and prognosis through reverse transcription PCR. They showed that the survival rate was significantly lower in stage III and unresectable stage IV CRC patients with CTCs positive for CD44v9 mRNA expression and speculated that CD44v9 mRNA in CTCs could be a useful marker to predict recurrence, prognosis, and treatment efficacy in CRC patients [162]. 

#### 3.1.2. CD47

CD47 is a cell surface ubiquitous glycoprotein, belonging to the immunoglobulin (Ig) superfamily. Upon binding to its ligand signal-regulatory protein α (SIRPα), expressed on macrophages and DCs, CD47 inhibits phagocytosis by these cell types [163]. For this reason, CD47 is known as a ‘don’t eat me signal’ [164] and its upregulation on CTCs might confer a nonimmunogenic profile, enabling them to escape from phagocytosis. An overexpression of the CD47 gene was found in CTCs from CRC patients as compared to corresponding primary tumor tissue, suggesting a potential survival advantage [153]. CD47 was also expressed in CD44^+^ CTCs from a progressive metastatic BC patient. The same patient had a primary tumor negative for CD47 but developed a bone metastasis with a high expression of CD47 after seven years; this suggests that CD47 expression was probably acquired during the initiation of metastatic dissemination [152]. 

#### 3.1.3. PD-L1

PD-1 is a member of the B7/CD28 family of co-stimulatory receptors. It regulates T cell activation through binding to its ligands, PD-L1 and PD-L2, both of which are expressed on many other cell types. When PD-L1 binds to PD-1, a strong inhibitory signal is transmitted into the T cell, leading to a reduction of cytokine production and suppression of T cell proliferation. Under physiological conditions, the PD-1/PD-L1 (or PD-1/PD-L2) interaction is necessary to mediate the natural immune tolerance [165]. In some tumors this protective mechanism is led to perversion through the overexpression of PD-L1 with the consequent prevention of an immune response against cancer [166]. The expression of PD-L1 has been demonstrated on CTCs of several malignancies [147,148,149,150,167,168,169,170,171,172] and associated with a poor prognosis [148,149,167,170].

#### 3.1.4. EpCAM

EpCAM is a cell surface glycoprotein that has gained considerable interest in the diagnosis and treatment of cancer because it is frequently overexpressed in epithelial tumors [173]. To date, EpCAM is the antigen of choice for CTC enrichment from patient blood samples and this principle is at the basis of the CS system, the only clinically-validated, FDA-cleared system for identification, isolation, and enumeration of CTCs from blood samples.

Although the available technologies are mostly EpCAM-dependent, the detractors of the use of this protein complain that a fraction of CTCs cannot be quantified yet through the CS (more aggressive, undifferentiated, or EMT cells?). The limits of current technologies for isolation of CTCs represent a hot topic, but their discussion is beyond the scope of our review. Other authors in this special issue, as well as ourselves elsewhere, have already extensively addressed these arguments [174]. For our purpose, it is enough to remember herein that, before arriving to patient’s bedside, a tumor marker must demonstrate its analytical and clinical validity and, finally, its clinical utility. The quality of available data determines the level of evidence, the strongest being Level 1, i.e., the definitive demonstration of clinical utility that can be obtained through a single, high-powered, prospective, randomized, controlled trial or from a meta-analysis or overview of multiple, well-designed studies. The European Pooled Analysis Consortium (EPAC) demonstrated the clinical validity of the CS assay, with Level 1 evidence in 2014 [5].

We should also remember that the meaning of any malignant feature of CTCs should be judged according to the degree of clinical validation of a certain phenotypical/molecular characteristic that we are using to identify CTCs in peripheral blood [175]. For this reason, after looking for potential targets in immunotherapy and their association with patient survival, we found and reported here, for the most, studies exploiting EpCAM-based technologies.

EpCAM overexpression has been associated with both decreased and increased survival of patients [176]. Dynamic changes in EpCAM expression frequently occur during tumor progression and its downregulation was observed during EMT [177]. Evidence suggests that epithelial plasticity could also be implicated in tumor immune escape [178]. In particular, the acquisition of an EMT phenotype has been associated with an inhibition of CTL-mediated tumor cell lysis in the human MCF-7 cell line [179]. Since EMT CTCs have also been correlated with disease progression and chemotherapy resistance [10,180]; enrichment systems that allow simultaneous investigation of both EpCAM^+^ and EpCAM^−^ CTCs are being employed in order to obtain more complete information about the role of EpCAM in tumor progression [181].

## 4. CTCs as Biomarkers in Cancer Immunotherapy

The progress achieved in recent years in understanding the molecular mechanisms underlying cancer has allowed the development of targeted therapies, with the hope to select the most appropriate treatment for individual patients. Among these strategies, immunotherapy has brought enormous progress to cancer treatment.

The main goal of cancer immunotherapy is to reinforce the patient’s suppressed immune system, ideally restoring its capability to eradicate cancer. The mechanisms of tumor escape from immune surveillance represent a ‘druggable’ Achilles’ heel for restoring immune control.

Cancer immunotherapy approaches can be passive or active. Passive immunotherapy is mainly used in case of weak or negative immune response and consists of ex vivo-activated cells or molecules that, once re-injected into the body, compensate for missing or deficient immune functions. This approach includes infusion of tumor-specific monoclonal antibodies (mAbs) directed against several targets (i.e., oncogenic pathways and osteoclast functions), infusion of cytokines, and adoptive cell transfer (ACT). Active immunotherapy strategies aim to stimulate in vivo a pre-existing immune response. To apply active immune-therapeutics, the patient’s immune system should be able to be competently stimulated and to mediate effector functions. This group includes vaccines, immune checkpoint inhibitors, and oncolytic viruses [182].

Since the detailed description of immunotherapeutic strategies goes beyond the aim of this review, we will focus our attention on the most successful treatments of solid tumors, in which CTCs have been investigated as biomarkers for patient monitoring (Table 1).

It is important to note here that most of the primary tumor targets listed below are not specific for CTCs alone. Indeed, some of them have already been detected as soluble markers or circulating tumor DNA (ctDNA) for a selection of tailored treatments, with lower costs and simpler execution as compared to CTC detection. For example, ctDNA was detected with a higher frequency than CTCs in metastatic BC [183]. However, while CTC numbers correlated with prognosis, baseline ctDNA levels did not. This suggests that ctDNA might be more useful in identifying mutations for therapeutic targets, rather than as prognostic biomarker [183].

### 4.1. Blocking of Oncogenic Pathways

Proteins involved in oncogenic pathways are often overexpressed during carcinogenesis and can be targeted to avoid the proliferation of tumor cells [184]. Tumor-specific mAbs induce tumor cell death by directly binding to tumor targets or stimulating antibody-dependent cellular cytotoxicity (ADCC) [185], complement-dependent cytotoxicity (CDC) [186], or antibody-dependent cellular phagocytosis (ADCP) [88]. Examples of mAbs directed against oncogenic pathways are anti-EGFR and anti-HER2.

#### 4.1.1. EGFR

The epidermal growth factor receptor (EGFR) is a transmembrane receptor with tyrosine kinase activity that, upon activation by EGF or other ligands, initiates mitogenic signaling across several pathways [187]. Overexpression of EGFR is associated with a more advanced disease and a more unfavorable prognosis; since it occurs in several malignancies, this pathway represents an ideal therapeutic target [188]. Cetuximab (Erbitux, Eli Lilly) was the first mAb prescribed to treat patients with advanced CRC expressing EGFR and was approved by the FDA in 2004. Thereafter, the FDA approved other anti-EGFR mAbs, either as single agents or in combination with other drugs. EGFR expression on CTCs has been demonstrated in patients with advanced breast, prostate, lung, and colorectal cancer by using the CS platform [189,190,191,192]. In CTCs from CRC patients, wide intra/interpatient variability in expression and gene amplification levels of EGFR was observed, which might explain differences in treatment response [193]. The persistence of CTCs, enriched and detected by AdnaTest from 38 advanced RAS-BRAF-wild-type CRC patients during treatment with cetuximab-irinotecan or panitumumab, was related to a decrease in OS and PFS [194]. Kuboki et al. demonstrated that a high CTC count assessed by CS predicted a decrease in OS in 63 patients with advanced CRC receiving cetuximab in combination with chemotherapy as third-line treatment, but EGFR expression in CTCs did not predict response to cetuximab [195].

#### 4.1.2. HER2

The human epidermal growth factor receptor 2 (HER2, also called HER2/neu or ERB-B2) is a transmembrane glycoprotein receptor with intracellular tyrosine kinase activity. Upon ligand binding, HER2 activates the PI3K/Akt signaling pathway, leading to cell proliferation and survival. HER2 gene amplification was initially reported in almost 30% of primary BC patients [196]. Trastuzumab (Herceptin, Genentech) is the first humanized IgG1-class mAb indicated for the treatment of HER2-amplified BC and was approved by the FDA in 1998.

HER2 expression on CTCs has been extensively tested in BC patients [197,198,199,200,201,202]. Notably, Meng and coworkers demonstrated in 24 patients with HER2-negative primary tumors that nearly 40% acquired a HER2 gene amplification in CTCs during cancer progression; they first obtained proof of concept that patients treated with a Herceptin-containing therapy had a partial or complete response [197]. Zhang et al. observed in 101 metastatic BC patients that HER2 status was different between CTCs and tumor tissues and that, in CTCs, it predicted the outcome of patients receiving anti-HER2 therapy. Indeed, about 62% of histologically HER2-positive patients had HER2-negative CTCs (detected through the CS system) at the time of sampling. Moreover, although all histologically HER2-positive patients received anti-HER2 therapy, the median PFS of HER2-positive CTC patients was significantly longer than that of HER2-negative CTC patients (8.5 vs. 3.5 months, *p* < 0.001) [202].

### 4.2. Osteoclast Regulation

Bone metastasis is common in solid tumors, particularly in breast and prostate cancer. It is the result of a complex process, in which tumor and immune cells participate by releasing cytokines and growth factors. The RANK/RANKL/osteoprotegerin (OPG) axis plays a key role in bone turn-over and is deregulated in many tumors. The differentiation and maturation of osteoclasts are mediated by the binding of RANKL to RANK. RANKL is produced by osteoblasts and stromal cells, while RANK is expressed on pre-osteoclasts. The RANK–RANKL interactions are tightly regulated by OPG, which acts as a soluble decoy receptor by preventing the binding of RANKL to RANK and blocking its activation, thereby inhibiting osteoclast genesis [203]. In a mouse model, tumor cells expressing RANK were shown to migrate to the bone, perhaps attracted by RANKL, which is abundantly expressed in this tissue [204]. Santini et al. demonstrated that RANK expression in primary BC is a predictive marker of bone metastasis occurrence and shorter skeletal DFS [205].

The RANK–RANKL interaction is a promising target for mAb immunotherapy in advanced cancer disease. Denosumab (Xgeva, made by Amgen Inc., and Prolia, made by Amgen, Inc.) is a human IgG2 mAb with a high affinity and specificity for human RANKL. By binding to RANKL, it prevents RANKL interaction with RANK (in a similar way to OPG), thus reducing the differentiation, activity, and survival of osteoclasts [206]. Xgeva and Prolia were approved by the FDA, respectively in 2010 and 2011, the former being indicated for the prevention of skeletal-related events in patients with bone metastases from solid tumors, and the latter for the treatment of bone loss in patients with prostate or breast cancer undergoing hormone ablation therapy. 

Concerning the expression of RANK on CTCs, Gray et al. analyzed circulating melanoma cells (CMCs) from 56 melanoma patients (40 late-stage and 16 early-stage) for RANK expression through multiparametric flow cytometry. RANK^+^ CMCs were detected in 22/40 late-stage and 4/16 early-stage patients. Interestingly, in two patients with 100% and 75% RANK^+^ CMCs, immunofluorescence staining of metastases, which had been removed one month prior to blood collection for CTC analysis, showed only a small fraction (2%) of RANK^+^ cells within the tumor. Furthermore, a change in CMC numbers after treatment start was not found, but the percentage of RANK^+^ CMCs increased after therapy with BRAF inhibitors and this was associated with a shorter PFS, whereas in patients treated with immune checkpoint inhibitors the increase in RANK^+^ CMCs was not apparent [43].

### 4.3. Immune Checkpoint Inhibitors

T cell activation is regulated at different levels during immune responses to prevent autoimmunity. The cytotoxic T-lymphocyte associated protein 4 (CTLA-4) and PD-1 immune checkpoint pathways play a key role in peripheral tolerance by operating at different stages of immune responses. CTLA-4 stops potential autoreactive T cells in the initial phase of naïve T cell activation, typically inside lymph nodes. Conversely, the PD-1 pathway regulates previously-activated T cells in the later stages of an immune response, primarily in peripheral tissues. As discussed above, cancer cells, which should be recognized and killed by T cells, have developed methods to evade the host’s immune system by exploiting peripheral tolerance [207]. 

#### 4.3.1. CTLA-4

CTLA-4 is a key inhibitor receptor that influences T cell function. In resting T cells, CTLA-4 is located in the intracellular compartment and is transported and expressed on the cell surface only after activation upon CD28 binding to B7-1 (CD80) and B7-2 (CD86) on APCs [208]. Once on the cell surface, the CTLA-4 inhibitory signal is transmitted through the binding of B7-1 and B7-2 on B cells and activated monocytes. Compared to CD28, CTLA-4 binds B7 molecules with a higher affinity and blocks further costimulation [209]. CTLA-4, therefore, downregulates T cell responses and APC function, resulting in immune tolerance [210].

Anti-CTLA-4 mAbs inhibit the binding of B7-1 or B7-2 on APCs to CTLA-4 on T cells. The consequent blockade of CTLA-4 signaling prolongs activation of T cells and restores their proliferation, thus amplifying T cell-mediated immunity and supporting the patient’s antitumor immune response [211]. In 2011, the FDA approved ipilimumab (Yervoy, Bristol-Myers Squibb) for the treatment of metastatic melanoma. To monitor the response to anti-CTLA-4 mAb treatments in melanoma patients, several immune circulating biomarkers have been evaluated and correlated to patient outcome (MDSCs, Tregs, CD3^+^, CD4^+^, and CD8^+^ T cells) [212,213,214,215].

Studies that use CTCs as biomarkers during anti-CTLA-4 mAb treatments are few. Khoja et al. and Klinac et al. employed CMCs as treatment response biomarkers in patients with melanoma receiving ipilimumab and/or other drugs, respectively using the CS and a manual multimarker immunomagnetic enrichment assay followed by microscopy quantification; however, in both cases the number of considered patients was insufficient to draw reliable conclusions [216,217]. Recently, Hong and collaborators isolated CMCs, by means of microfluidic enrichment, from 16 metastatic melanoma patients undergoing therapy with ipilimumab, and developed a CTC scoring assay to evaluate a 19-gene digital RNA signature. They showed that the use of this quantitative CTC score applied to the serial monitoring of patients was predictive of long-term response to immunotherapy, thus offering an alternative to the analysis of repeated tumor biopsies, which are invasive and insufficiently precise to guide new or ongoing treatments [218]. However interesting as these approaches are, to clarify the role of CMCs/CTCs in the monitoring of patients treated with anti-CTLA-4 mAbs, further studies are needed in large, well-defined subgroups of patients undergoing the same treatment regimen.

#### 4.3.2. The PD-1/PD-L1 Axis

As discussed above, a tumor overexpressing PD-L1 protects itself from T cells, leading to exhaustion and neutralization. The PD-1/PD-L1 inhibitors prevent the PD-1/PD-L1 interaction, thus facilitating an efficacious immune response against the tumor. Clinical studies indicated that antibodies that block PD-1 and PD-L1 have a reliable effect on many advanced malignancies [219]. The PD-1 blockers pembrolizumab (Keytruda, Merck & Co., Inc., Kenilworth, NJ, USA) and nivolumab (Opdivo, Bristol-Myers Squibb Company, New York, NY, USA) were approved by the FDA in 2014 for patients with advanced melanoma and have subsequently been approved for other cancer types. 

PD-L1 can be detected by immunohistochemistry on tumor or immune cells; however, the utility of this marker in predicting which patients might benefit from immune checkpoint inhibitors is controversial [220]. Indeed, since most PD-L1 positive tumors are not affected by anti-PD-1/PD-L1 therapy, the predictive value of PD-L1 in tumor biopsies is so low that it is unacceptable to use as a biomarker in treatment selection [221]. Furthermore, a discordant PD-L1 expression between primary tumors and metastases was observed [222]. 

Mazel and colleagues first reported the expression of PD-L1 in CTCs from 16 patients with HR^+^, HER2^-^ metastatic BC, as assessed by the CS platform. CTCs expressing PD-L1 ranged from 0.2 to 100% and PD-L1 intensity varied between different patients and between CTCs within the same sample. The authors supposed that PD-L1^+^ CTCs might be able to escape the immune system control and, therefore, represent a target for anti-PD-L1 therapies [147]. 

To understand whether CTCs that express PD-L1 could represent a predictive biomarker during anti-PD-1 therapy, Nicolazzo and colleagues investigated the expression of PD-L1 in CTCs from 24 patients with metastatic NSCLC treated with nivolumab and deduced that it assumes a predictive significance after several months from the beginning of the therapy [148]. Recently, many papers have been published in which the expression of PD-L1 on CTCs has been studied in patients undergoing immunotherapy [148,149,168,169]. Despite the different CTC enrichment and enumeration platforms and PD-L1 expression assays, the majority of these studies agree in affirming that a high PD-L1^+^ CTC burden is associated with worse OS and PFS [148,149,167,170]. However, it seems premature to draw definitive conclusions because of the low number of analyzed patients.

In Section 4.3.1, we cited the paper by Hong et al. describing a novel method based on a digital RNA signature and a CTC scoring assay to predict patient response to ipilimumab. Noteworthy, the authors also successfully tested their CTC score on samples from 33 melanoma patients receiving pembrolizumab, hence showing the potential clinical validity of this use of CMCs/CTCs as a noninvasive biomarker in cancer immunotherapy [218].

### 4.4. Adoptive Cell Transfer (ACT)

ACT has been proven to be an effective immunotherapeutic method for the treatment of cancer and has achieved promising results in anticancer clinical trials [223]. One of the first anticancer ACT therapies was based on infusion of tumor infiltrating lymphocytes (TILs) for the treatment of melanoma [224] and obtained an objective response rate of 49–72% when the preparative chemotherapy-induced lymphocytic depletion was performed before TIL infusion [225]. Other ACT-based approaches use CTLs, NK cells, genetically-engineered lymphocytes expressing highly active TCRs, and chimeric antigen receptors (CARs) [226]. In NK cell-based immunotherapy, some studies have shown a decrease in the number of CTCs, which appears to be associated with treatment efficacy and positive patient outcome [227,228,229]. In particular, Liang et al. compared two groups of patients with recurrent BC, the first (*n* = 18) treated with autogeneic NK cells and the second (*n* = 18) with allogeneic NK cells. In the group treated with allogeneic NK cells, they found that the number of CTCs, enriched by immunomagnetic sorting and counted by FACS, decreased significantly (from 13.13 ± 5.83 before treatment to 6.88 ± 4.95 one month after the final transfusion, *p* = 0.01). In contrast, changes in CTC levels in the group treated with autogeneic NK cells were not significant (*p* > 0.05) [227]. Using the same enrichment and counting systems, CTCs have also been studied to evaluate allogeneic NK cell-based ACT in stage IV NSCLC patients (*n* = 31). Lin et al. observed a decrease in CTC numbers, from 27.12 ± 9.286 one day before treatment to 14.02 ± 5.872 at 30 days after the treatment [228]. Qin et al. also observed a decrease in CTC counts in stage II-IV hepatic carcinoma patients (*n* = 16) one month after immunotherapy with allogeneic NK cells [229].

### 4.5. Cancer Vaccines

A large number of therapeutic vaccines against cancer have shown only little effect, perhaps due to the use of non-mutated self antigens (unable to mediate effective antitumor responses), monovalent antigen-targeting strategies that may select resistant tumor variants, or suboptimal delivery systems that resulted in weak and short-lived antigen-specific T cell responses. A further obstacle is represented by a highly immunosuppressive tumor microenvironment [230]. 

The identification and validation of predictive biomarkers that accurately reflect immune responses in tissues, including tumors, will be important future tasks of the Human Vaccines Project [231]. Some studies have already been published on the use of CTCs for this purpose. Stojadinovich et al. detected CTCs (by CS) in patients without clinical evidence of BC but at high risk of recurrence. Patients were immunized with the E75-peptide vaccine and a significant reduction in both total CTCs and HER2/neu^+^ CTCs was demonstrated over the course of vaccination. These preliminary data suggest that enumeration of CTCs might serve as a surrogate marker to monitor the immunologic response to BC-targeting vaccines [232]. 

### 4.6. Immunotherapies Targeting CTCs

We have seen that CTCs were proven to be useful prognostic markers and that researchers are working assiduously to define their predictive utility. In this last paragraph, we report some preliminary studies on immunotherapies against CTCs. These studies, performed in vitro or in murine models, give an idea of the variety of targets and strategies that can be considered in cancer therapy.

The anti-CD44 antibody RG7356 prevented tumor cell adhesion to hyaluronic acid in vitro and caused tumor growth inhibition in vivo in xenograft models using cell lines expressing the CD44s isoform [233]. It has also been demonstrated that CD44-targeting in xenografts results in tumor cell phagocytosis mediated by macrophages. It has been supposed that the RG7356-based treatment induces an upregulation of cytokines and chemoattractants that recruit and activate TAMs [234]. Although TAMs mainly promote tumor progression, it has recently been shown that they are able to phagocytize tumor cells in the presence of mAbs targeting specific tumor antigens [235]. 

In primary human bladder cancer, a marked expression of CD47 was seen in CD44^+^ tumor initiating cells (TICs) that escape phagocytosis. In the same study, an anti-CD47 mAb restored the ability of macrophages to phagocytize TICs in vitro [236]. 

Monoclonal and recombinant antibodies have been used to directly target EpCAM^+^ cells [237,238,239,240]. A recombinant vaccinia virus expressing full-length EpCAM (VV GA733-2) was able to promote macrophage-mediated ADCC of antigen-positive CRC targets in a murine model [241]. Although promising results were obtained in preclinical studies, the efficacy of EpCAM-directed mAbs and of VV GA733-2 still remains to be demonstrated through large clinical trials [242].

Other preliminary studies could have interesting implications in CTC research. Gül et al. showed in mice inoculated with B16F10 melanoma cells that unstimulated Kupffer cells could bind, but not eliminate tumor cells circulating into the liver; interestingly, when stimulated with a tumor-specific, anti-gp75 mAb, Kupffer cells efficiently removed tumor cells in an ADCP-mediated manner. Phagocytosis of tumor cells was dependent on both the high-affinity IgG-binding Fcγ receptor I (FcγRI) and on the low-affinity FcγRIV, and resulted in inhibition of liver metastasis [88]. Mitchell et al. developed peripheral blood leukocytes coated with liposomes previously conjugated with E-selectins and TNF-related apoptosis inducing ligand (TRAIL). When mice intravenously inoculated with tumor cells were treated with the functionalized leukocytes, intercepted cancer cells in the circulation underwent TRAIL-mediated apoptosis. Since the cytotoxic activity of these leukocytes resembled that of NK cells, they were named ‘unnatural killer cells’ [243]. In conclusion, these two examples may represent promising therapeutic strategies to improve the immune antitumor intervention in CTC-positive cancer patients.

## 5. Future Opportunities

Compared with the scenario delineated at the end of the last century, modern oncology has made an epochal breakthrough in patient treatment: the findings of molecular oncology now allow selection of the best treatment for individual patients at any time during disease evolution, maximizing the results and minimizing the side effects. Immunotherapy represents a further improvement, because it permits manipulation of the immune system in order to stimulate recognition and elimination of cancer cells, leaving intact normal surrounding tissues.

However, as in all the best stories, evil is lurking and promises to complicate matters. In our case, the challenge is represented by heterogeneity and plasticity of the tumor that, under pressure from treatment with a targeted drug, is able to evade towards alternative, yet-unexplored pathways, or to favor the growth of a minority of tumor cells insensitive to the ongoing treatment. The only option, in this not-so-unusual case, is to recognize as early as possible the incoming resistance and to change treatment.

Alternatively, we should combine different drugs to target, at the same time, different tumor cell subsets at the lowest dose sufficient to reduce tumor burden without inducing resistance and limit side effects of the drug combination. The cooperative support of immunotherapy, focused on eliminating damaged cancer cells, also promises, in principle, a successful eradication of microscopic groups of tumor cells. The utility of this kind of therapeutic strategy needs to undergo the proof of facts, in ad hoc designed clinical studies based on a robust group of biomarkers.

For the reason explained in the introduction, we believe that CTCs are a good point of observation of tumor evolution and research on the interplay between CTCs and the immune system might be useful to control tumor growth and, hopefully, block it.

By reviewing the literature, we showed that most cell populations of the immune system interact with CTCs, conditioning their shedding from the primary tumor, survival, homing, or even their further growth. In principle, the ambiguous relationship between CTCs and the immune system, with its anti- or protumor functions depending on the context, might be a resource rather than a problem if we are able to manipulate the immune system to our scope. The main lesson is that any interaction with the immune system as well as any characteristic of CTCs might be transformed into an Achilles’ heel of the tumor.

In the near future, the knowledge on the metastatic mechanisms that we are gaining from studies on CTCs will allow the implementation of a growing number of immunotherapeutic strategies—some of these options are already real. For example, in 2004, Mosolits et al. identified a promising anti-EpCAM vaccine: they treated 13 CRC patients with an anti-EpCAM vaccine (*n* = 7) or an anti-idiotypic antibody mimicking EpCAM (*n* = 6) in combination with GM-CSF. They detected a long-lasting EpCAM-specific proliferative T cell response in the first group compared to the second [244]. In a more recent work, Choi and coworkers showed that vaccines based on DCs pulsed with EpCAM peptides elicit a strong antigen-specific CTL response and result in a significant suppression of tumor growth in a mouse model. This study, performed with the human hepatoma cell line HepG2, suggests that peptides from cancer stem cells (CSCs) might be a source of antigens for vaccination-based immunotherapy, with the aim to eliminate the CSCs responsible for tumor relapse [245].

On the other hand, since the EMT process is thought to play a role in tumor progression and therapy resistance, immunotherapies directed against tumor cells undergoing EMT are being investigated. The vaccines directed against brachyury, a transcription factor that is associated with tumor EMT, represent an example (Table 1). As reviewed in detail in the work by Hamilton and colleagues, these vaccines are an attractive therapeutic strategy to be used in combination with other treatments [246].

Finally, research on CTCs and immunotherapy of tumors will soon benefit from the extraordinary advances recently achieved in the fields of genomics and transcriptomics, at both a molecular and a computational level. High-throughput technologies, such as next-generation sequencing (NGS), and computational tools for data analysis are now available to study tumor escape mechanisms and to unveil still unknown interactions between tumor and immune cells [247]. Strikingly, these technologies can also be applied to CTCs. For example, single-cell RNA-Seq is already being used to investigate origin, phenotype, and drug resistance pathways of CTCs [248,249].

It has recently been reported that a new procedure, based on the collection of a large volume of peripheral blood (the Diagnostic Leucapheresis), increases the total number of collected CTCs up to 30 fold, thus promising to make high-throughput technologies usable tools for the great majority of patients [250].

Future possible applications of these tools could allow, for instance, the discovery of new interactions between CTCs and the immune system; potentially-immunogenic neoantigens on CTCs in order to target them and/or the primary tumor by means of immunotherapy; neoantigens on CTCs that might reduce the success rate of immunotherapies recognizing only the original antigen (but not the mutated form); other escape mechanisms on CTCs that are shared with the primary tumor; and specific molecular targets on CTCs (rather than on biopsies) to identify and stratify patients who would benefit from immunotherapy. As demonstrated with the work by Hong et al. (which we discussed above), such innovative approaches can also allow us to follow CTC dynamics and reinforce the predictive value of CTCs as a biomarker for serial monitoring of patients during immunotherapies and other treatments [218].

Thus, by combining cancer immunotherapy with molecular and computational genomic tools applied to CTCs, we could move personalized diagnosis and therapy of cancer to the next level.

## 6. Conclusions

By reviewing the main studies that directly or indirectly address the relationships between the immune system and CTCs we could observe that this is a new frontier of cancer research with a promising impact in the clinics.

However, concerning the feasibility of a prompt transfer from lab benchtop to patient bedside of the concepts discussed in these pages, it seems opportune to introduce two cautionary notes. First, the number of patients analyzed in the majority of the studies discussed here is very low; second, often, the experimental evidence obtained in in vitro or murine models has never been tested in clinical trials. It is clear that further studies in homogeneous groups of patients, who are undergoing the same treatment regimen and for whom a comprehensive characterization of their disease is available (including molecular markers and considering primary tumor, CTCs, and possible metastases), are necessary before we will be able to fully exploit the translational potential of CTC research in the oncoming era of cancer immunotherapy.

Finally, the recent molecular and computational genomic tools are gaining increasing attention thanks to the huge quantity of novel information they can provide to researchers. Over the next few years, we will probably witness a revolutionary progress in oncology and CTC-aided immunotherapy of tumors.

## Figures and Tables

**Table 1:** List of clinical trials where CTCs are being/have been investigated in patients undergoing cancer immunotherapies. The list is based on the ClinicalTrials.gov database as of August 2018.

Trial Number	Official Title	Study Type	Status	Phase	Estimated/Actual Enrollment	Disease	CTC/Other Biomarker Measurem. Method	Outcome Measures/Objectives	Sponsor/ Collaborators	Start/ Completion Date	Publications
NCT03515798	A Prospective Multicenter Open-label, Randomized Phase II Study of Pembrolizumab in Combination with Neoadjuvant (F)EC–Paclitaxel Regimen in HER2-negative Inflammatory Breast Cancer	Int	Not yet recruiting	Phase 2	81	Inflammatory breast cancer	NP	Evaluation of: pathological CR and DLT rates, IDFS, EFS, and OS; PD-L1 expression in pre-, per-, and post-treatment tissue. Occurrence of adverse events. Measurement of baseline CTCs for prospective validation of their prognostic value in IBC; disease monitoring (ctDNA sequencing). Identification of mechanisms of treatment resistance (immune profiling, NGS, and mouse xenografing)	Institut Paoli-Calmettes; MSD France	June 2018–April 2025	NP
NCT03213041	I-CURE-1: A Phase II, Single Arm Study of Pembroluzimab Combined with Carboplatin in Patients with Circulating Tumor Cells (CTCs) Positive Her-2 Negative Metastatic Breast Cancer (MBC)	Int	Recruiting	Phase 2	100	Metastatic breast cancer	CellSearch, CellSieve, sequencing	Evaluation of: PFS, OS, ORR, CBR, immune-related response, immune-related clinical benefit, TTNM in CTC-positive mBC patients previously treated with anthracyclines and taxanes undergoing treatment with carboplatin–pembrolizumab; ORR and CBR in relation to PD-L1 expression in tissue and CTCs. Measurement of: PD-L1 in CTCs and immune cells such as CAMLs and their correlation with therapeutic benefit. Measurement of ctDNA and sequencing analysis of TCR and their correlation with CTC enumeration and therapeutic benefit	Northwestern University; Merck Sharp & Dohme Corp.; National Cancer Institute (NCI)	September 2017–July 2022	NP
NCT03434912	Identification and Evaluation of the Potential Biomarkers on Circulating Tumor Cells and Tumor Related Rare Cells in Cancer Patients Undergoing Immunotherapy	Obs	Recruiting	NP	80	NP	NP	Isolation and analysis of CTCs and tumor-related rare cells before and after immunotherapy. Identification of potential biomarkers associated with clinical outcome. Association between changes of rare tumor cells and clinical outcome	MiCareo Taiwan Co., Ltd.; Taipei Veterans General Hospital; Taiwan	May 2017–April 2020	NP
NCT03070002	A Phase II, Open Label Study to Evaluate Denosumab in Patients with ER and/or PR-Positive, HER2-Negative Metastatic Breast Cancer (MBC) With Bone Metastases and Detectable Circulating Tumor Cells (CTCs)	Int	Recruiting	Phase 2	42	Metastatic breast cancer	NP	Assessment of: the effect of denosumab in patients with bone metastases and ≥5 CTCs who are in PR or SD after starting systemic therapy by measuring the fraction of patients with reduction in CTCs; the effect of denosumab on CTC enumeration considered as a continuous variable (percent change from baseline); median-PFS through statistical analysis evaluating the relationship between longitudinal CTC counts and PFS; the effect on CTC profiling and characterization of stem cell phenotype (CTC-EMT); the type of PD. Analysis of RANKL expression	Northwestern University; Amgen; National Cancer Institute (NCI)	March 2017–March 2022	NP
NCT02978118	Exploring Relevant Immune-based Biomarkers and Circulating Tumor Cells During Treatment with Immune Checkpoint Inhibitors in Genitourinary Malignancies	Obs	Recruiting	NP	10	Metastatic renal cell carcinoma and metastatic urothelial carcinoma	NP	Evaluation of: change in the number of T cells, B cells, myeloid cells before and after treatment with immune therapies; number of patients with detectable CTCs; prevalence of TILs and TAMs at baseline; change in CTCs over time; distribution of CTC difference scores across tumor response categories of CR, PR, SD, and PD	Duke University; University of Wisconsin, Madison	March 2017–March 2019	NP
NCT02948985	Evaluation of Individual Peripheral Blood Circulating Tumor Cells Combined with Tumor Marker Detection of Efficacy of Chemotherapy in Patients with Advanced Colorectal Cancer: An Observational Clinical Trial	Obs	Not yet recruiting	NP	100	Metastatic colorectal cancer	SE-iFISH, BEAMing	Correlation of: RAS status on CTCs with clinical outcome of patients with histologically-confirmed RAS and B-Raf wild-type mCRC treated with FOLFIRI ± cetuximab; mutations in ctDNA with cetuximab resistance	Shanghai General Hospital; Shanghai Jiao Tong University School of Medicine	January 2017–December 2019	NP
NCT03114631	Single-center Trial Evaluating the Safety and Efficacy of MUC-1/WT-1 Peptide or Tumor Lysate-pulsed Dendritic Cell Immunotherapy for the Patients with Pancreatic Cancer	Int	Recruiting	Phase 1 and phase 2	30	Pancreatic cancer	NP	Assessment of: number of participants with PR or CR at 1 year; number of participants who survived at 1, 2, 3 years or more; immune response (increase of antigen-specific T cells, decrease of Tregs and other cell subsets associated with tumor progression); CTC count (decrease of EpCAM^+^ CD45^-^ CTCs)	The Republican Research and Practical Center for Epidemiology and Microbiology; Belarusian state medical university	January 2017–December 2019	NP
NCT02456571	Defining the Relevant Immune Checkpoints Expressed on Metastatic Prostate Cancer Circulating Tumor Cells	Obs	Recruiting	NP	40	Metastatic prostate cancer	CellSearch	Evaluation of: change in expression of PD-L1, PD-L2, B7-H3, and CTLA-4 on CTCs of four groups of patients undergoing different treatments; change over time in mutational profiles, AR-variant expression and immune/tumor-related RNA signatures in CTC-enriched blood; expression (prevalence) of PD-L1, PD-L2, B7-H3, and CTLA-4 in metastatic tumor tissue obtained by elective CT or US-guided research biopsies in up to 10 patients and comparison of this expression percentage with CTC immune checkpoint expression	Duke University; Janssen Research & Development, LLC	November 2016–June 2019	NP
NCT02933255	Phase I/II Study of PROSTVAC in Combination with Nivolumab in Men With Prostate Cancer	Int	Recruiting	Phase 1 and phase 2	29	Prostate cancer	NP	Evaluation of: safety and changes in T cell infiltration in the tumor after neoadjuvant treatment; change in peripheral PSA-specific T cells in patients treated with PROSTVAC and nivolumab; intraprostatic Treg infiltration with CD4 and FOX-P3 stainings; PSA changes secondary to vaccination, including rate of biochemical recurrence after prostatectomy; MRI changes secondary to treatment; changes in PD-L1 expression; pathologic responses (including pathologic CR); changes in immune cell subsets in the periphery; changes in soluble immune-mediating factors (such as cytokines, etc.) in sera; changes in CTC levels in mCRPC cohort	National Cancer Institute (NCI); National Institutes of Health Clinical Center (CC)	October 2016–August 2021	NP
NCT02915445	T Cells Armed with Chimeric Antigen Receptor Recognizing EpCAM for Patients With Nasopharyngeal Carcinoma and Breast Cancer	Int	Recruiting	Phase 1	30	Nasopharyngeal carcinoma or breast cancer	NP	Evaluation of: number of participants with treatment-related adverse events/dose-limiting toxicity as assessed by CTCAE v4.0; RR of participants treated with EpCAM^+^ CAR-T cells assessed by RECIST v1.1; persistence of EpCAM^+^ CAR-T cells and correlation with the RR; persistence of EpCAM^+^ CTCs	Sichuan University	July 2016–July 2019	NP
NCT02827344	Feasibility Study of PD-L1 Expression Analysis on Circulating Tumor Cells by Immunocytochemistry and MDSCs Level Evolution Analysis in Non-small Cell Lung Cancer Treated With PD-L1 or PD1 Inhibitor	Obs	Unknown status	NP	51	Stage IV non-small cell lung cancer	ISET, immunocytochemistry	Assessment of: feasibility of analysis of PD-L1 expression on CTCs; percentage of CTCs expressing PD-L1 after 4 cycles of immunotherapy; evolution of MDSC counts in response to treatment	University Hospital, Toulouse	October 2015 –October 2016	[168]
NCT02552394	Anti-Prostate-Specific Membrane Antigen Monoclonal Antibody J591 in Patients with Advanced Prostate Cancer and Unfavorable Circulating Tumor Cell Counts	Int	Recruiting	Phase 1	24	Metastatic prostate cancer	CellSearch	Assessment of: decrease of CTC count in mPCa patients with elevated baseline CTC counts undergoing mAb Hu-J591 treatment with different dose levels; PSA decline rate across all dose levels; 89Zr-DFO-huJ591 PET/CT imaging both pre- and post-mAb hu-J591 infusion to describe objective imaging responses; duration of biochemical and/or measurable disease response through PSA dosage and/or CT scans; number of participants with treatment-related adverse events as assessed by CTCAE v4.0; overall and prostate cancer specific survival rate of subjects following treatment; pain change from baseline as reported on the brief pain inventory questionnaire	Weill Medical College of Cornell University	July 2015–July 2019	[251]
NCT02510781	A Study on Neoadjuvant Therapy for Her-2 Positive Breast Cancer and the Prognosis by Detecting Circulating Tumor Cells	Int	Unknown status	Phase 2	200	Stage II–III breast cancer	NP	Assessment of: pCR rate; clinical response rate; number of adverse events	Hospital Affiliated to Military Medical Science, Beijing	January 2015–December 2017	NP
NCT02179515	An Open Label Phase I Study to Evaluate the Safety and Tolerability of a Modified Vaccinia Ankara (MVA) Based Vaccine Modified to Express Brachyury and T-Cell Costimulatory Molecules (MVA Brachyury-TRICOM)	Int	Completed	Phase 1	38	Lung, breast, prostate, ovarian tumors (others)	NP	Evaluation of: safety and tolerability of escalating doses of MVA-brachyury-TRICOM vaccine; CD8^+^ and CD4^+^ cell immunologic response as measured by an increase in brachyury-specific T cells; evidence of clinical benefit (such as PFS), RECIST criteria, reduction in serum markers, and/or reduction in CTCs; frequency of immune cell subsets in peripheral blood and changes in serum levels of cytokines and Abs to brachyury; correlation of brachyury expression in tissue samples with clinical outcomes	National Cancer Institute (NCI); National Institutes of Health Clinical Center (CC)	June 2014–February 2018	[252,253]
NCT01804465	A Randomized Phase 2 Trial of Immediate vs. Delayed Anti-CTLA4 Blockade Following Sipuleucel-T Treatment for Prostate Cancer Immunotherapy	Int	Recruiting	Phase 2	54	Prostate cancer	NP	Evaluation of: impact of the timing of ipilimumab administration on PAP/PA2024-specific immune responses by SipT; PSA measurements; radiographic clinical responses; modulation of effector and regulatory T cells; safety of combining ipilimumab with SipT; CTC count; T cell gene and microRNA signatures	University of California, San Francisco; M.D. Anderson Cancer Center; Bristol-Myers Squibb; Dendreon	January 2014–December 2019	NP
NCT02412462	Phase I Dose Escalation Study of AB-16B5 in Subjects with an Advanced Solid Malignancy	Int	Completed	Phase 1	15	Advanced solid malignancies	NP	Assessment of: number of participants with an adverse event; plasma concentrations of AB-16B5; objective tumor responses in subjects with measurable disease according to RECIST. Monitoring of EMT and stem cell biomarkers in CTCs and paired tumor biopsies	Alethia Biotherapeutics	April 2015–January 2017	NP
NCT01975142	Validity of HER2-amplified Circulating Tumor Cells to Select Metastatic Breast Cancer Considered HER2-negative for Trastuzumab-emtansine (T-DM1) Treatment	Int	Active, not recruiting	Phase 2	155	Metastatic breast cancer considered HER2 negative on primary tumor	FISH, IF	Assessment of: tumor response rate to T-DM1 in patients with HER2-amplified CTCs; detection rate of HER2-amplified CTCs; heterogeneity rate between CTCs and correlations with patient characteristics; technical failure rate and reproducibility of HER2 FISH on CTCs; correlation between HER2 FISH and IF on CTCs; PFS; disease control rate (responsive and stable cases); correlation between treatment efficacy and HER2 FISH results; changes in CTC numbers during treatment; ctDNA before and during treatment; toxicity	Institut Curie	October 2013–November 2017	[254]
NCT02450448	The Detection of Circulating Tumor Cells (CTCs) in Patients with Renal Cancer Undergoing Cryosurgery Combined With DC-CIK Treatment	Obs	Completed	NP	60	Stage II-IV renal cancer	Multiparameter FCM and RT-PCR	Enumeration of CTCs after cryosurgery in patients receiving or not receiving DC-CIK treatment	Fuda Cancer Hospital, Guangzhou	June 2013–December 2015	NP
NCT02450435	The Detection of Circulating Tumor Cells (CTCs) in Patients with Prostatic Cancer Undergoing Cryosurgery Combined With DC-CIK Treatment	Obs	Completed	NP	60	Stage II-IV prostatic cancer	Multiparameter FCM and RT-PCR	Enumeration of CTCs after cryosurgery in patients receiving or not receiving DC-CIK treatment	Fuda Cancer Hospital, Guangzhou	June 2013–December 2015	NP
NCT02450422	The Detection of Circulating Tumor Cells (CTCs) in Patients with Colorectal Cancer Undergoing Cryosurgery Combined With DC-CIK Treatment	Obs	Completed	NP	60	Stage II-IV colorectal cancer	Multipa-rameter FCM and RT-PCR	Enumeration of CTCs after cryosurgery in patients receiving or not receiving DC-CIK treatment	Fuda Cancer Hospital, Guangzhou	June 2013–December 2015	NP
NCT02450357	The Detection of Circulating Tumor Cells (CTCs) in Patients with Breast Cancer Undergoing Cryosurgery Combined With DC-CIK Treatment	Obs	Completed	NP	60	Stage II–IV breast cancer	Multipa-rameter FCM and RT-PCR	Enumeration of CTCs after cryosurgery in patients receiving or not receiving DC-CIK treatment	Fuda Cancer Hospital, Guangzhou	June 2013–December 2015	NP
NCT02416635	The Detection of Circulating Tumor Cells (CTCs) in Patients with Liver Cancer Undergoing Cryosurgery Combined With DC-CIK Treatment	Obs	Completed	NP	60	Stage II–IV liver cancer	Multipa-rameter FCM and RT-PCR	Enumeration of CTCs after cryosurgery in patients receiving or not receiving DC-CIK treatment	Fuda Cancer Hospital, Guangzhou	June 2013–December 2015	NP
NCT02412384	The Detection of Circulating Tumor Cells (CTCs) in Patients with Lung Cancer Undergoing Cryosurgery Combined With DC-CIK Treatment	Obs	Completed	NP	120	Stage II–IV lung cancer	Multipa-rameter FCM and RT-PCR	Enumeration of CTCs after cryosurgery in patients receiving or not receiving DC-CIK treatment	Fuda Cancer Hospital, Guangzhou	June 2013–February 2016	NP
NCT02406846	The Detection of CTCs in Patients with Pancreatic Cancer Undergoing Cryosurgery Combined With DC-CIK Treatment	Obs	Completed	NP	80	Stage II–IV pancreatic cancer	Multipa-rameter FCM and RT-PCR	Enumeration of CTCs after cryosurgery in patients receiving or not receiving DC-CIK treatment	Fuda Cancer Hospital, Guangzhou	June 2013–December 2015	NP
NCT01548677	TRastuzumab in HER2-negative Early Breast Cancer as Adjuvant Treatment for Circulating Tumor Cells (CTC) (“TREAT CTC” Trial)	Int	Completed	Phase 2	1317	HER2 negative primary breast cancer	NP	Detection of CTCs. Comparison of CTC detection rate at week 18 between the trastuzumab treatment arm and the observational arm. Evaluation of: RFI, IDFS, DFS, and OS; feasibility, reliability, within-patient reproducibility, and variability of CTC assays; correlation of CTC detection rate with RFI, IDFS, DFS, and OS; cardiac safety of trastuzumab treatment	European Organisation for Research and Treatment of Cancer—EORTC; Hoffmann-La Roche; Janssen Diagnostics, LLC; SUCCESS; UNICANCER	April 2013–March 2017	[255]
NCT01640444	Randomized Phase II Study to Explore the Influence of BRAF and PIK3K Status on the Efficacy of FOLFIRI Plus Bevacizumab or Cetuximab, as First Line Therapy of Patients with RAS Wild-type Metastatic Colorectal Carcinoma and <3 Circulating Tumor Cells	Int	Active, not recruiting	Phase 2	240	Metastatic colorectal cancer	NP	Evaluation of: PFS, OS, RR, and R0 surgery rate; baseline CTC count and its correlation to PFS, OS, and RR; adverse events; correlation of molecular status of biomarkers related to cellular and tumoral reproduction and/or mode of action and PFS, OS, and RR	Spanish Cooperative Group for the Treatment of Digestive Tumours (TTD); Roche Pharma AG	July 2012–November 2018	NP
NCT01640405	Phase III, Randomized Clinical Trial to Evaluate FOLFOX + Bevacizumab Versus FOLFOXIRI + Bevacizumab as First Line Treatment of Patients with Metastatic Colorectal Cancer Not Previously Treated and With Three or More Circulating Tumoral Cells	Int	Active, not recruiting	Phase 3	350	Metastatic colorectal cancer	NP	Evaluation of: PFS, OS, RR, and R0 surgery rate; baseline CTC count and its correlation to PFS, OS, and RR; correlation of RAS, BRAF, and PI3K mutations and PFS, OS, and RR; adverse events; correlation of molecular status of biomarkers related to cellular and tumoral reproduction and/or mode of action and PFS, OS, and RR	Spanish Cooperative Group for the Treatment of Digestive Tumours (TTD); Roche Phara AG	July 2012–November 2018	NP
NCT01185509	A Phase II, Single Arm, Open Label Study to Evaluate the Efficacy and Safety of Trastuzumab and Vinorelbine in Advanced Breast Cancer Patients with HER2 Negative Primary Tumors and HER2 Positive Circulating Tumor Cells	Int	Terminated	Phase 2	31	HER2 negative primary breast cancer	NP	Evaluation of: ORR, CBR, PFS, and CTC levels. Description of CTC number and CTC characteristics before and after therapy, and correlation of these findings with response; safety and tolerability of trastuzumab and vinorelbine treatment	Dana-Farber Cancer Institute; Brigham and Women’s Hospital; Beth Israel Deaconess Medical Center; Massachusetts General Hospital; Genentech, Inc.	November 2010–September 2017	NP
NCT01456065	A Phase I, Open, Randomized, Study to Investigate the Safety of Active Immunotherapy with Fully Mature, TERT-mRNA and Survivin—Peptide Double Loaded Dendritic Cells (DCs) in Subjects With Advanced Epithelial Ovarian Cancer, Enrolled in the Study Within Twelve Weeks After Completing Primary Therapy	Int	Unknown status	Phase 1	15	Ovarian epithelial cancer	NP	Assessment of: incidence of adverse events and clinical relevant deviations from laboratory parameters; number of CTCs prior to vaccination and at follow up; number of autologous DCs loaded with tumor-specific antigens; time to progression (CA-125 and CT); OS; immune monitoring prior to vaccination and during treatment;	Life Research Technologies GmbH	September 2010–April 2013	[256]
NCT00879866	An Open-label, Phase Ib, Dose-escalation Trial on the Safety, Tolerability, Pharmacokinetics, Immunogenicity, Biological Effects and Antitumor Activity of EMD 521873 in Combination with Local Irradiation (20 Gy) of Primary Tumors or Metastases in Subjects With Non-small Cell Lung Cancer Stage IIIb With Malignant Pleural Effusion or Stage IV With Disease Control (Partial Response or Stable Disease) After Application of 4 Cycles of Platinum-based, First-line Chemotherapy	Int	Completed	Phase 1	15	Non-small cell lung cancer	NP	Assessment of: safety, tolerability, and MTD (if reached) with EMD 521873 doses of up to 0.45 mg/kg; PK of EMD 521873 in combination with local tumor irradiation; immunogenicity of EMD 521873 in combination with local tumor irradiation by measuring the induction of anti-EMD 521873 Abs; best overall response; changes in tumor marker levels and CTC numbers after treatment; best overall response after second-line therapy and duration of the response; PFS and OS; biological/immune responses following treatment by assessing changes in relevant parameters including leukocyte subset analysis and molecular markers of immune activation (e.g., cytokines/chemokines, IL-2 receptor, and neopterin)	Merck KGaA	April 2009–September 2012	[257]
NCT00924092	Open Label Phase I Study to Evaluate the Safety and Tolerability of Vaccine (GI-6207) Consisting of Whole, Heat-Killed Recombinant Saccharomyces Cerevisiae Genetically Modified to Express CEA Protein in Adults with Metastatic CEA-Expressing Carcinoma	Int	Completed	Phase 1	25	Prostate, breast, lung, colorectal, head and neck cancer	NP	Determination of: safety and tolerability of escalating doses of a heated-killed yeast-based vaccine targeting tumors that express CEA; evaluation of: CD4^+^ and CD8^+^ cell immunologic response; humoral immune response to yeast antigen; evidence of clinical benefit such as PFS, OR; decreases in CTCs; tumor markers	National Cancer Institute (NCI); National Institutes of Health Clinical Center (CC)	March 2009–August 2012	[258]
NCT02048540	Phase 2 Study of Neoadjuvant Bevacizumab Plus DOF Versus DOF in Local Advanced Gastric Carcinoma and Its Association with Circulating Tumor Cell	Int	Completed	Phase 1 and phase 2	86	Stage IIIb-IIIc gastric carcinoma	NP	Assessment of: R0 resection rate; pCR rate; OS; DFS; ORR; safety of perioperative treatment and surgery; CTC number change before and after therapy	Chinese PLA General Hospital	February 2009–December 2013	NP
NCT00429247	A Pilot Randomized Phase II Study of Adjuvant Administration of Trastuzumab (HERCEPTIN) Versus Observation After the Completion of Adjuvant Chemotherapy and Radiotherapy in Patients with Stage I-III Breast Cancer Who Have Detectable Disseminated and/or Circulating Tumor Cells (DTCs and/or CTCs) in the Bone Marrow or/and the Peripheral Blood Before or/and After the Completion of Adjuvant Treatment	Int	Completed	Phase 2	75	Stage I-III operable breast cancer	NP	Comparison of disease-free interval of patients with early-stage breast cancer. Evaluation of CK19 mRNA-positive CTC elimination	University Hospital of Crete	February 2003–December 2007	NP

Abbreviations: androgen receptor (AR); cancer-associated macrophage-like cells (CAMLs); beads, emulsion, amplification, magnetics (BEAMing); cancer antigen (CA); cell-free circulating tumor deoxyribonecleic acid (ctDNA); circulating tumor cells (CTCs); clinical benefit rate (CBR); common terminology criteria for adverse events (CTCAE); complete response (CR); computed tomography (CT); dose-limiting toxicity (DLT); dendritic cell (DC); cytokine-induced killers (CIK); disease-free survival (DFS); docetaxel oxaliplatin 5-FU CF (DOF); epithelial-to-mesenchymal (EMT); event-free survival (EFS); flow cytometry (FCM); 5-FU/LV + irinotecan (FOLFIRI); interventional (int); immune-related complete response (irCR); immune-related partial response (irPR); immune-related RECIST (irRECIST); immunofluorescence (IF); inflammatory breast cancer (IBC); invasive disease-free survival (IDFS); isolation by size of tumor cells (ISET); maximum tolerated dose (MTD); metastatic breast cancer (mBC); metastatic colorectal cancer (mCRC); metastatic prostate cancer (mPCa); myeloid-derived suppressor cells (MDSCs); not provided (NP); observational (obs); overall response rate or objective response rate (ORR); overall survival (OS); partial response (PR); pathological complete remission (pCR) rate; pharmacokinetics (PK); positron emission tomography (PET); progressive disease (PD); progression-free survival (PFS); prostate specific antigen (PSA); radical (R0) resection; recurrence-free interval (RFI); response evaluation criteria in solid tumors (RECIST); response rate (RR); stable disease (SD); subtraction enrichment and immunostaining-fluorescence in situ hybridization (SE-iFISH); time to new metastases (TTNM); trastuzumab-emtansine (T-DM1); tumor-associated macrophage (TAM); tumor-infiltrating lymphocyte (TIL); ultrasound (US).

## Abbreviations

ACT	adoptive cell transfer
ADCC	antibody-dependent cellular cytotoxicity
APC	antigen-presenting cell
BC	breast cancer
CAML	cancer-associated macrophage-like cell
CAR	chimeric antigen receptor
CDC	complement-dependent cytotoxicity
CMC	circulating melanoma cell
CRC	colorectal cancer
CS	CellSearch
CSC	cancer stem cell
CTC	circulating tumor cell
CTL	cytotoxic T lymphocyte
CTM	circulating tumor microemboli
DC	dendritic cell
DCreg	regulatory dendritic cell
DFS	disease-free survival
ECM	extracellular matrix
EGFR	epidermal growth factor receptor
EMT	epithelial-to-mesenchymal transition
EpCAM	epithelial cell adhesion molecule
FAS	first apoptosis signal receptor
FDA	Food and Drug Administration
G-CSF	granulocyte-colony stimulating factor
HER2	human epidermal growth factor receptor 2
ILC2	type 2 innate lymphoid cell
mAb	monoclonal antibody
MAM	metastasis-associated macrophage
MHC	major histocompatibility complex
MDSC	myeloid-derived suppressor cell
MET	mesenchymal-to-epithelial transition
MTF	macrophage–tumor cell fusion
NET	neutrophil extracellular trap
NK (cell)	natural killer (cell)
NSCLC	non-small cell lung cancer
OS	overall survival
PBMC	peripheral blood mononuclear cell
PD-L1	programmed death-ligand 1
PFS	progression-free survival
PMo	patrolling monocyte
RANKL	nuclear factor kappa-B ligand
TAM	tumor-associated macrophage
tBreg	tumor-evoked regulatory B cell
TCR	T cell receptor
TDSF	tumor-derived soluble factor
Th (cell)	T helper (cell)
TIL	tumor-infiltrating lymphocyte
TLR	Toll-like receptor
Treg	regulatory T lymphocyte
VCAM-1	vascular cell adhesion molecule-1
